# Antidiabetic effects of betulinic acid mediated by the activation of the AMP-activated protein kinase pathway

**DOI:** 10.1371/journal.pone.0249109

**Published:** 2021-04-05

**Authors:** Tae-Jun Song, Choon-Ho Park, Kyu-Ree In, Jong-Bae Kim, Joo Hee Kim, Miran Kim, Hye Jin Chang

**Affiliations:** 1 Graduate School of Life Science, Handong Global University, Pohang, South Korea; 2 Graduate School of Clinical Pharmacy and Pharmaceutics, Ajou University, Suwon, South Korea; 3 Department of Life Sciences, College of Natural Sciences, Ajou University, Suwon, South Korea; 4 College of Pharmacy, Ajou University, Suwon, South Korea; 5 Department of Obstetrics and Gynecology, Ajou University Hospital, Ajou University School of Medicine, Suwon, South Korea; Max Delbruck Centrum fur Molekulare Medizin Berlin Buch, GERMANY

## Abstract

Betulinic acid (BA) is a naturally arising pentacyclic triterpenoid that has anti-malarial, anti-retroviral, anti-inflammatory, and anti-cancer biological effects. More recently, it has been reported to possess anti-obesity activity mediated by the activation of AMP-activated protein kinase (AMPK). We further investigated antidiabetic activity of BA in mouse tissues at the cellular and systemic levels. We found that BA stimulated AMPK in a similar fashion to the known AMPK activators, such as 5-aminoimidazole-4-carboxamide-1-β-D-ribofuranoside and metformin. Notably, the level of glucose uptake by BA was not altered by wortmannin, suggesting that this activation did not depend on phosphoinositide 3-kinase. Furthermore, BA diminished blood glucose levels in alloxane-treated ICR mice and in untreated mice during the glucose tolerance test. BA also stimulated mRNA expression of glucose transporter 4, which could partly explain increased glucose uptake. BA also increased AS160 phosphorylation by insulin-independent mechanisms in the extensor digitorum longus muscle. These results indicate that BA may serve as a promising therapeutic agent for diabetes by activating AMPK, like metformin. Notably, BA also enhanced mouse endurance capacity, indicating that it also affects metabolic regulation in addition to its antidiabetic activity.

## Introduction

At the present time, there are over 150 million people suffering from diabetes mellitus (DM) worldwide, and the patients is expected to increase to over 300 million by 2025. Type 2 diabetes (T2D) mostly account for these global pandemic [1,2]. DM refers to the disorders that are characterized by elevated blood glucose level [1,3]. According to both clinical stages of hyperglycemia, i.e., high levels of circulating glucose, and the etiological types, the disease was classified as type 1 and type 2 diabetes, it was accepted by the World Health Organization and the American Diabetes Association [3]. Over the last 30 years, a large number of studies have been focused to understanding the pathophysiology of type 2 DM [4]. Hyperglycemia is a typical and serious symptom in T2D [5]. As insulin resistance is a major pathophysiologic abnormality of T2D, insulin-sensitizing agents that could restore insulin resistance and control hyperglycemia in T2D patient, has been interested in therapeutic approach [6].

Until now, pharmacologic target of insulin resistance is mainly directed to two pathways: peroxisome-proliferator-activating receptors (PPARs) [7] and AMP-activated protein kinase (AMPK) [8]. Thiazolidinediones and biguanides are the worldwide used agents in T2D treatment field. Although thiazolidinediones are commonly used, they have undesirable side effects, such as weight gain, fluid retention, and heart failure. The biguanide, metformin does not cause weight gain but mainly acts in the liver rather than in muscles, and it also causes side effects such as nitric acidosis, diarrhea, nausea, and vomiting [9]. Therefore, there is a global need to find a better medicine without side effects for T2D [6]. Traditional, complementary, and alternative medicines are useful sources for novel drug development [10]. For example, investigators have structurally modified natural guanide product, came from *Galega officinalis* (goat’s rue) and produced metformin, which is a biguanide derivative of guanide, to improve drug’s efficacy. A merits of traditional medicines is that they have been used to treat human diseases from for many years, so there is considerable knowledge concerning their safety and effects on the human body. However, in most cases, there is little rigorous scientific evidence proving their efficacy, and the mode of their action is often unknown. To overcome these problems, it is essential to identify individual biologically active components of traditional medicines and investigate their specific effects in well-defined biological systems and animal models translationally relevant to humans.

In this study, we have investigated antidiabetic effects of the naturally occurring compound betulinic acid (BA), a pentacyclic lupane-type triterpenoid abundant in plants. A various biological activities of triterpenoid has been known, including antiviral, antitumor, and anti-inflammatory actions [11–14]. More recently, we have reported that BA has an anti-obesity effect mediated by the activation of AMP-activated protein kinase (AMPK), suggesting therapeutic potential of BA in the treatment of the metabolic syndrome [15]. We also noted that mistletoe, which has been traditionally used in antidiabetic therapy for a long time, contains large amounts of BA [16]. It is well known that mistletoe extract improves biomarkers associated with DM [13,16,17]. Therefore, we sought to investigate the potential antidiabetic efficacy of BA.

AMPK is a ubiquitously expressed serine/threonine protein kinase that is activated by depletion of phospho-compounds which is high energy source in cellular level. It is sensor of intracellular fuel status [18]. Activated AMPK initiates a complex signaling to produce energy, they facilitate the uptake and oxidation of important substrates for ATP synthesis and decreases in ATP-consuming processes, such as protein, lipid, and glycogen synthesis [19]. Activation of AMPK has also regulated the glucose transport [20]. However, the related pathway are indefinable. Recently, it has been shown that triterpenes, including BA, stimulate glucose uptake and glycogen synthesis via the activation of AMPK in insulin-resistant human HepG2 cells [21]. To our knowledge, it was the first report that demonstrated an antidiabetic effect of plant triterpenes. However, a detailed mechanism of the antidiabetic efficacy of triterpenes has not been elucidated [21].

In this study, we sought to establish the molecular mechanism of the antidiabetic effect of BA. First, we investigated how BA affected AMPK phosphorylation. In addition, we investigated the effects of BA on the glucose transporter expression, glucose uptake, and blood glucose concentration in experiments with mouse tissues and cells. Furthermore, we compared BA effects to those of the known antidiabetic drug 5-aminoimidazole-4-carboxamide riboside (AICAR). Finally, we examined the effect of BA on the overall metabolic regulation by measuring the endurance capacity of mice exercised on a treadmill.

## Materials and methods

### Reagents

DMEM, penicillin/streptomycin, and fetal bovine serum (FBS) were purchased from GIBCO. Krebs-Henseleit prebuffer was purchased from Sigma. Anti-phospho AMPKα and anti-AS160 (a 160-kDa substrate of the Akt Ser/Thr kinase) antibodies were purchased from Cell Signaling (S1 Fig). Horseradish peroxidase (HRP)-conjugated anti-goat IgG, anti-mouse IgG, and anti-rabbit IgG antibodies were purchased from Jackson ImmunoResearch Ltd. Nitrocellulose membranes and X-ray films for western blot were purchased from Pall Corporation, Intron, AGFA, respectively. Supersignal West Pico chemiluminescent substrate and West Femto chemiluminescent substrate were purchased from Pierce. SYBR Green master mix for real-time PCR was purchased from Applied Biosystems.

### Cells

Mouse myoblast C2C12 cells obtained from American Type Cell Culture (ATCC) were grown as a monolayer in Dulbecco’s Modified Eagle’s Medium (DMEM) supplemented with 10% FBS and 1% penicillin/streptomycin. For myotube differentiation, when C2C12 cells were at 90% confluence, growth medium was changed to DMEM supplemented with 2% horse serum and 1% penicillin/streptomycin and cells were incubated for a week. The C2C12 cells differentiated rapidly, and produced characteristic contractile myotubes. To detect of mycoplasma contamination, e-Myco Mycoplasma PCR detection kits were used.

### Animals

Specific pathogen-free male ICR mice and C57Bl/6J mice aged 7 weeks were obtained from the Dae-Han Laboratory Animal Center, Republic of Korea. All animal experiments were underwent according to the protocol approved by the Institutional Animal Care and Use Committee of Han Dong Global University. These mice were took cared in the Laboratory of Animal Experiments of the Institute of Bioscience at the Han Dong Global University under clean conditions. Subject mice were free to eat foods and water *ad libitum*. Each group was composed of five mice for experiments. The mice were housed in in a conventional environment, with a temperature of 22° to 24°C, and a relative humidity of 40% to 60%, under a 12/12-hour light/dark cycle. After the end of the experiment, the mice were euthanized in the CO2 chamber.

### Treadmill test

Exercise capacity was measured on a treadmill (Columbus Instruments, Columbus, OH) contained in a Plexiglas’s chamber equipped with an impact grid behind the belt to keep the animal running during the test. The impact grid delivered 0.2mA shock, which was uncomfortable, but did not physically harm or injure the animals, and the mouse were acclimated to the test conditions before the experiment. During the course of the test, the mice ran for 10 minutes on a treadmill with 5 slopes at a speed of 15 m/min. The speed was then gradually increased from 15 to 25m/min and remained at 25m/min until exhausted. When the mouse was unable to run for 10 seconds, electric shocks were stopped.

### Immunoprecipitation

We performed immunoprecipitation of extensor digitorum longus muscle lysate (300 μg protein) with 2 μg of anti-AS160 antibody at 4°C overnight. The 20 μL of protein G agarose (GE Healthcare) added to muscle lysate for 3h at 4°C and subsequently washing with lysis buffer was carried out three times, and following four times with PBS. The immunocomplex was boiled in NuPAGE LDS sample buffer (Invitrogen) and subjected to sodium dodecyl sulphate-polyacrylamide gel electrophoresis.

### Muscle incubation procedures

After mice were anesthetized and extensor digitorum longus muscles were quickly removed. And muscles tissues were incubated in Krebs-Henseleit prebuffer (Sigma) at 30°C saturated with a gas mixture containing 95% O_2_ and 5% CO_2_. BA (10–100 μg/mL, TCI, Japan), AICAR (2 mmol/L; Toronto Research Chemicals, Toronto, Canada), wortmannin (500 nmol/L; Sigma), insulin (60 nmol/L Actrapid; Novo Nordisk, Bagsværd, Denmark), and/or DMSO (0.01%; Sigma) were added to the prebuffer for subsequent incubations.

### Cell lysate preparation

The cultures were collected for western blotting to detect AMPK and β-actin expression levels. After being washed three times with PBS, the cells were resuspended in 120 μL of cell lysis buffer (PRO-PREP cell lysis buffer, iNtRON). After scraping the cells by using a cell scrapper, the samples were boiled for 10 min. This was followed by sonication after which, the samples were centrifuged at 13,000 rpm for 10 min at 4°C, and the supernatants were collected. The protein contents of cell lysate were determined by a bicinchoninic acid (BCA) assay kit (Pierce).

### Western blot analysis

Equal quantities of cell lysate were loaded onto a 10% polyacrylamide gel and blotted on a nitrocellulose membrane (Pall Corporation) in transfer buffer (25 mM Tris, 192 mM glycine, 20% methanol) at 100 V, in 70 min after electrophoresis. The membrane was blocked with 10% milk for 2 h at room temperature, and then incubated for 2 h at room temperature with antibodies against phospho-AMPKα (1:1,000), phospho-AS160 (1:1,000), and β-actin (1:2,000). The membranes were washed with PBST (0.1% Tween-20 in PBS) three times and then incubated for 1 h at room temperature with HRP-conjugated secondary antibodies (1:10,000). After washing the membranes with PBST three times, the signals were detected with Supersignal West Pico and West Femto Chemiluminescent substrate using an X-ray film (AGFA).

### Real-time PCR analysis

After cell harvesting, total RNA was isolated from the samples with an Easy-spin RNA extraction kit (Intron, Korea). Template cDNA was generated from 5 μg of total RNA using Superscript (Invitrogen). In a fluorescent thermal cycler (LightCycler; Roche Diagnostics Ltd, Lewes, UK), 10 percent of each RT reaction was amplified in a 20 μL PCR mix containing 4 pmol of each primer, 1× SYBR Green master mix, an 4 mM MgCl_2_. Samples were incubated in the LightCycler for the initial denaturation at 94°C for 30 s, followed by 30 PCR cycles. Each cycle consisted of denaturation at 95°C for 15 s, annealing at 55°C for 32 s, and primer extension 72°C for 32 s. Endogenous *Actb* (β-actin) signal was used as internal control. To confirm amplification of specific transcripts, at the end of each PCR procedures, melting curve profiles were produced by cooling the sample to 65°C for 15 s and heating it slowly to 95°C with continuous measurement of fluorescence. The following oligonucleotide primers were used: peroxisome proliferator-activated receptor gamma coactivator 1-alpha (*Ppargc1a*) forward (F): TCG/ATG/TGT/CGC/CTT/CTT/GC; *Ppargc1a* reverse (R): ACG/AGA/GCG/CAT/CCT/TTG/G, *Slc2a1* (F): CTC/CAT/CAT/GGG/CAA/TGC/AG; *Slc2a1* (R): GGC/AGA/AGG/GCA/ACA/GGA/TAC, *Slc2a4* (F): AAC/CAG/CAT/CTT/CGA/GTC/GG; *Slc2a4* (R): CGA/GAC/CAA/GGT/GAA/GAC/CG, *Actb* (F): CCA/TCC/TGC/GTC/TGG/ACT/TG; *Actb* R: TTC/CCT/CTC/AGC/TGT/GGT/GG.

### Measurement of blood glucose levels in alloxan-treated hyperglycemic mice

Alloxan (70 mg/kg, Sigma, St. Louis, MO) was injected into ICR mice via the tail vein. On the next day after alloxan injection, 200 μL of DEAE fractions (0.5 M and 1 M) were injected intraperitoneally at different concentrations (400 μg/mL, 200 μg/mL, 100 μg/mL, 50 μg/mL). After 10 days, repeated intraperitoneal injection of DEAE fractions was performed. Blood of mice was taken through a heparinized capillary tube. Blood glucose level was measured using a glucose measurement kit (Pharmacia). Water consumption was assessed by volume.

### Glucose transport assay

C2C12 myotubes were exposed to BA dissolved in complete DMEM containing 1% FBS for 24 h. Cell monolayers were rapidly washed in Krebs Ringer Phosphate (KRP) at pH 7.4 and incubated with or without 20 μM cytochalasin B for 10 min at 37°C. Insulin (100 nM) or vehicle treatment was for 10 min, and this was followed by treatment with ^3^[H]-2-deoxyglucose (0.2 mM, 0.2 μCi) for 10 min. Glucose uptake was terminated by three times washing with ice-cold PBS. Cells were suspended in 0.1% sodium dodecyl sulfate solution, and radioactivity counts were taken.

### Metabolic studies

For the glucose tolerance test, mice were pretreated with BA intraperitoneally after an overnight fast. After 1 h, glucose at a dose of 2 g/kg body weight was injected intraperitoneally, and blood glucose was monitored using blood glucose strips and a One Touch Ultra glucometer (Johnson and Johnson) at indicated times. For the insulin tolerance test, mice were fasted for 6 h and were pretreated with BA (200mg/kg) by intraperitoneal injection. After 1 h, insulin (0.2 U/kg body weight) was injected intraperitoneally, and blood glucose levels were measured at indicated times. Insulin tolerance test data are presented as the percentage of initial blood glucose concentration.

### Statistical analysis

Mean ± standard error means (SEM) were calculated and one-way analysis of variance in conjunction with the post-hoc Tukey’s multiple comparison tests were conducted to determine the significance of differences between experimental groups. Differences were considered significant if *P* < 0.05. SPSS for Windows, version 22.0. (SPSS Inc., Chicago, Ill., USA) was utilized for all statistical tests.

## Results

### BA augments AMPK phosphorylation in C2C12 cells

**Fig 1:**
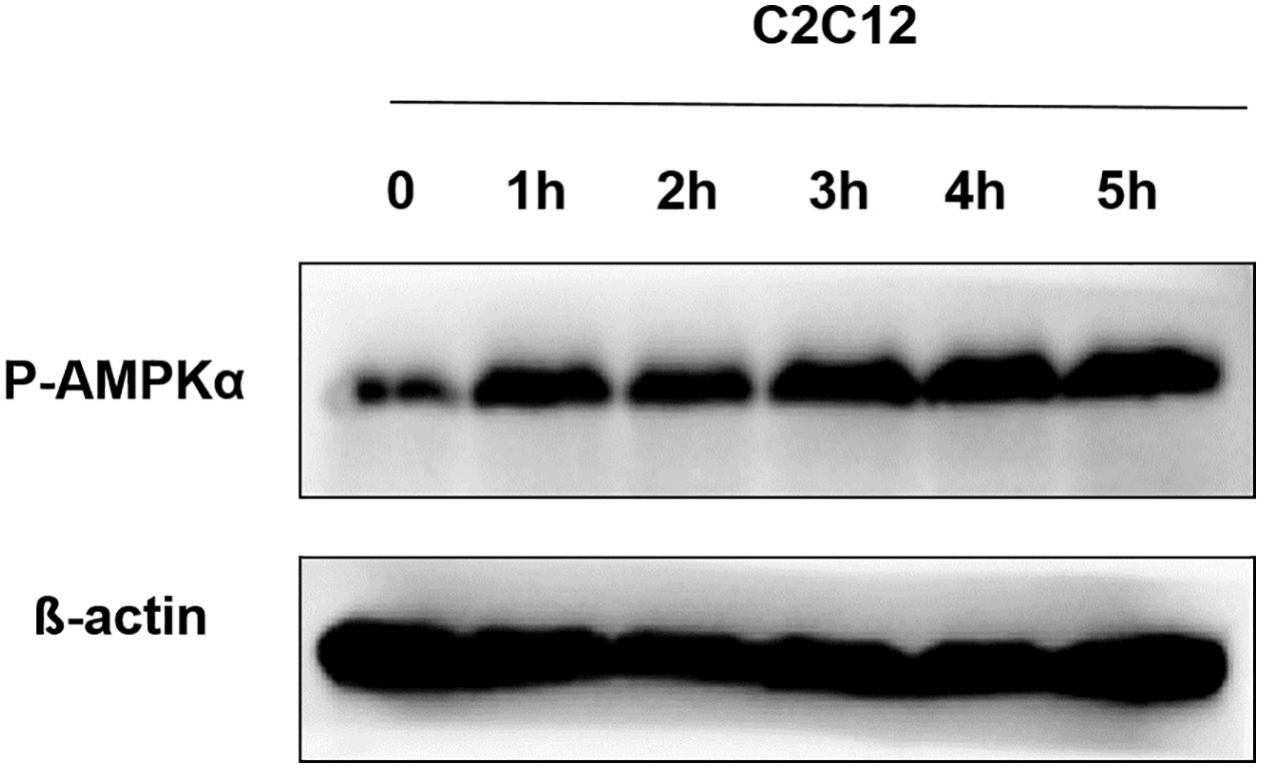
Effect of BA on AMPK phosphorylation in C2C12 cells. After C2C12 cells were treated with 100 μg/mL BA for various periods of time, AMPK phosphorylation levels were detected by western blot. The levels of β-actin expression served as loading control.

To elucidate the molecular mechanisms of the antidiabetic effect of BA, we first investigated whether it activates AMPK, one of the key metabolic sensor kinases. Muscle is a main organ for utilization of glucose. The exposure of C2C12 cells derived from mouse myoblasts to BA induced an increase of AMPK Thr172 phosphorylation as measured by western blot time-dependent manner (Fig 1). Thr172 residue localizes in the active site of the AMPK-α subunit and its phosphorylation, which reached the maximum level at 1 h after the treatment with 100μg/mL BA, correlates with the overall level of enzyme activity.

### BA decreases blood glucose level in alloxan-treated ICR mice

**Fig 2:**
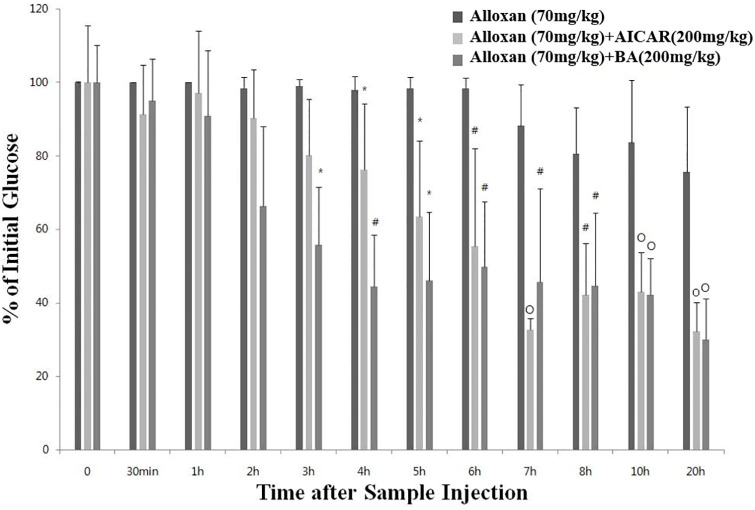
Acute effects of BA on blood glucose level in alloxan-treated ICR mice. Acute blood glucose test results in ICR mice treated with alloxan (70 mg/kg), alloxan (70 mg/kg) + AICAR (200 mg/kg), or alloxan (70 mg/kg) + BA (200 mg/kg). The values represent the mean ± standard error of the mean (*N* = 5 for each group). Statistical significance of differences from the control alloxan (70 mg/kg) treated group is indicated as follows: *P < 0.05, #*P* < 0.01, and o*P* < 0.001.

We hypothesized that as an AMPK activator, BA could affect blood glucose level in alloxan-treated mice. Alloxan is a chemical agent that selectively destroys insulin-producing pancreatic β cells in rodents and many other animal species [22]. Supplementation of alloxan-treated mice with intraperitoneally injected BA improved the acute blood glucose levels, which started to decrease in 2 h following the BA injection, reducing significantly compared to that of mice in the control alloxan-only group between 3 and 20 h post injection (Fig 2).

### BA increases insulin sensitivity in alloxan-treated ICR mice

**Fig 3:**
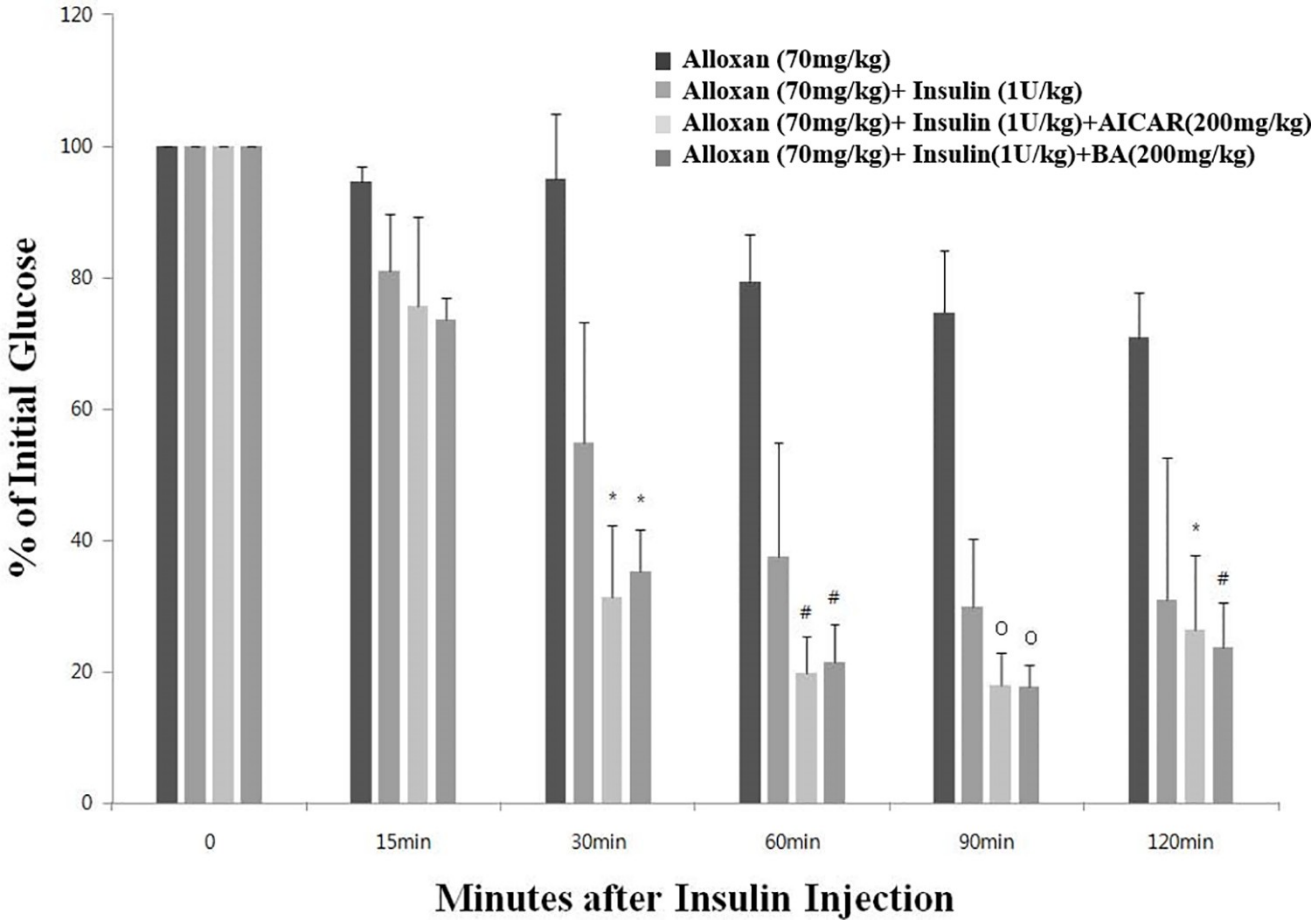
Increased insulin sensitivity of alloxan-treated ICR mice following their treatment with BA. Results of insulin tolerance test in mice treated with alloxan (70 mg/kg), alloxan (70 mg/kg) + insulin (1 U/kg), alloxan (70 mg/kg) + insulin (1 U/kg) + AICAR (200 mg/kg), or alloxan (70 mg/kg) + insulin (1 U/kg) + BA (200 mg/kg). The values represent the mean ± standard error of the mean (*N* = 5 for each group). Statistical significance of differences from the control alloxan (70 mg/kg) + insulin (1 U/kg) group is indicated as follows: *P < 0.05, #*P* < 0.01, and o*P* < 0.001.

**Fig 4:**
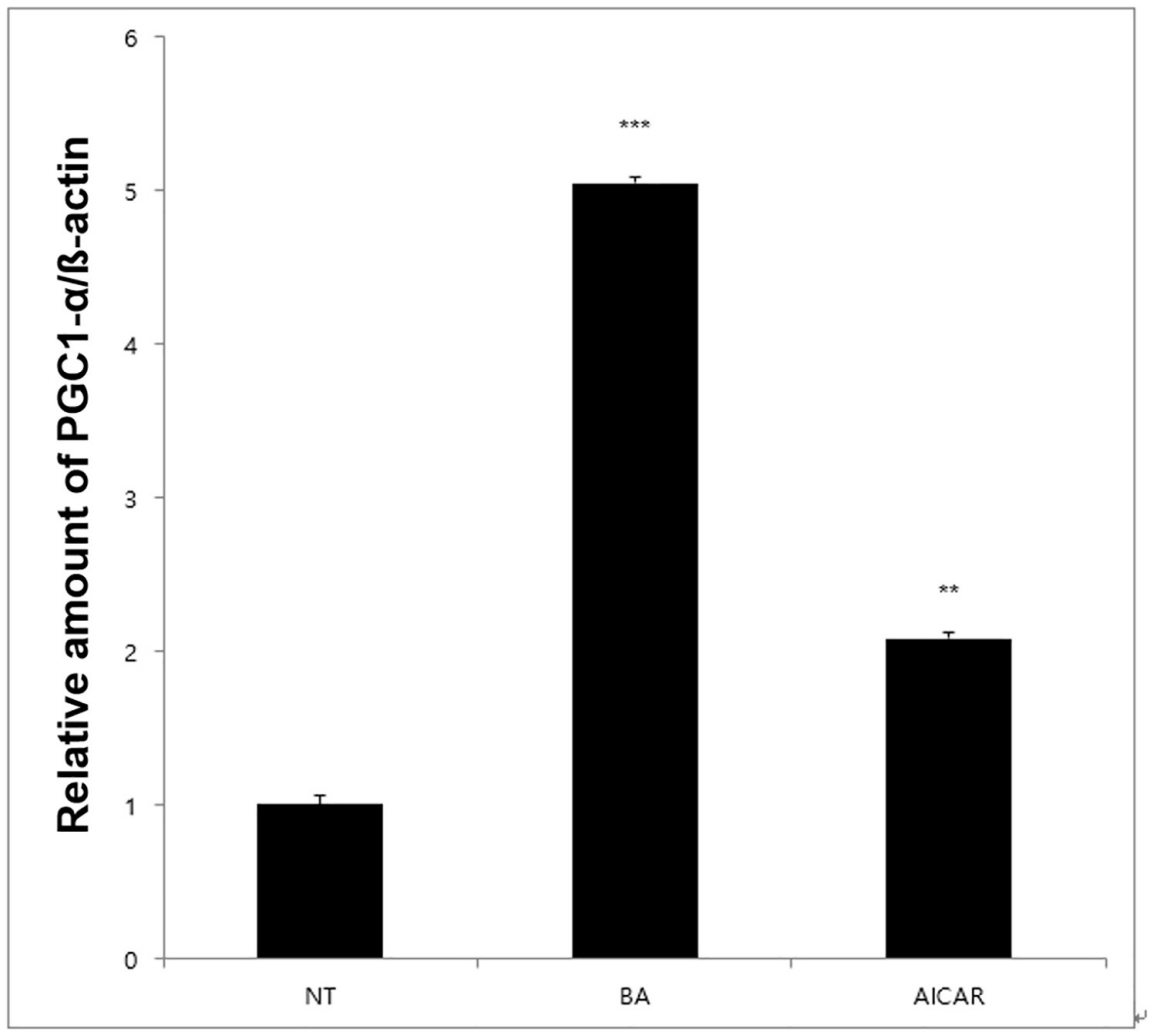
Effect of BA on *Ppargc1a* mRNA expression. Real-time PCR demonstrated that treatment with BA and AICAR increased mRNA expression of *Ppargc1a* that encodes PGC1-α protein, a marker of insulin sensitivity in skeletal muscle. The values represent the mean fold change ± standard error of the mean (*N* = 3). Statistical significance of differences from the control non-treated group (NT) is indicated as follows: **P < 0.01, ***P < 0.001.

To investigate the effect of BA on insulin sensitivity, we measured blood glucose level in diabetic mice injected with BA 1 h before insulin treatment. As shown in Fig 3, blood glucose levels in mice treated with insulin and either AICAR or BA were lower than those in insulin-only group. To further investigate the mechanism of elevated insulin sensitivity, we measured mRNA levels of the *Ppargc1a* gene, which encodes the insulin sensitivity marker PGC1-α, and found that they were highly increased by BA treatment in C2C12 cells (Fig 4). Therefore, BA may increase insulin sensitivity by upregulating PGC1-α expression.

### Increased glucose tolerance in BA-treated ICR mice

**Fig 5:**
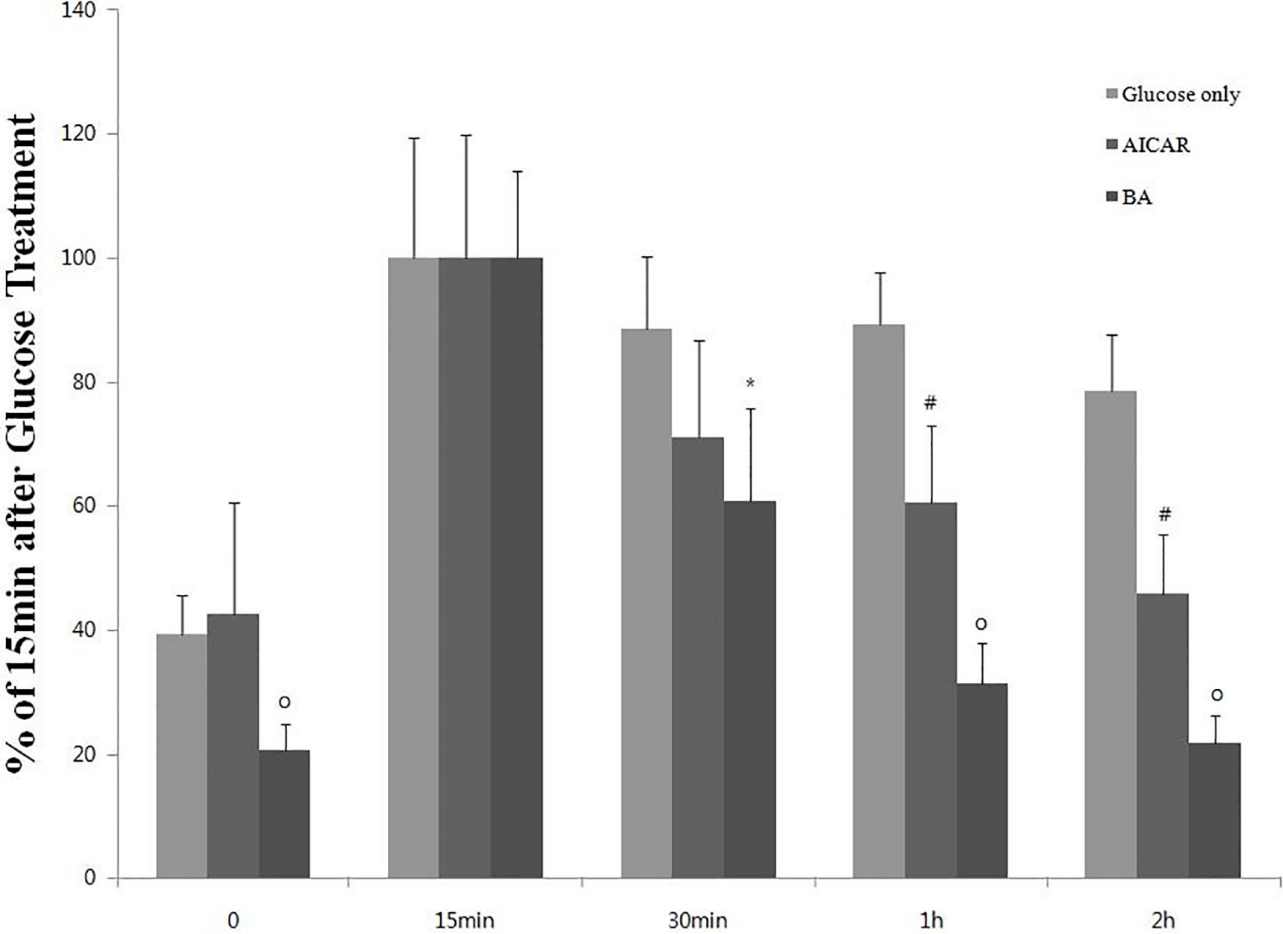
Effect of BA on glucose level following acute glucose challenge in ICR mice. Results of the glucose tolerance test in ICR mice that were treated with glucose (2 g/kg), glucose (2 g/kg) + AICAR (200 mg/kg), or glucose (2 g/kg) + BA (200 mg/kg). The values represent the mean ± standard error of the mean (*N* = 5 for each group). ’% of 15 min after glucose treatment’ means the relative percentage amount of blood glucose when the blood glucose level measured 15 minutes after glucose injection is set to 100%. Statistical significance of differences from the control glucose-only group is indicated as follows: *P < 0.05, #*P* < 0.01, and o*P* < 0.001.

To investigate the effect of BA on glucose tolerance, we measured blood glucose level in ICR mice, which received an injection of BA 1 h before acute glucose challenge. As shown in Fig 5, blood glucose levels of mice pretreated with AICAR or BA were lower than those of mice that received glucose only.

### BA upregulates *Slc2a4* mRNA expression in C2C12 cells

**Fig 6:**
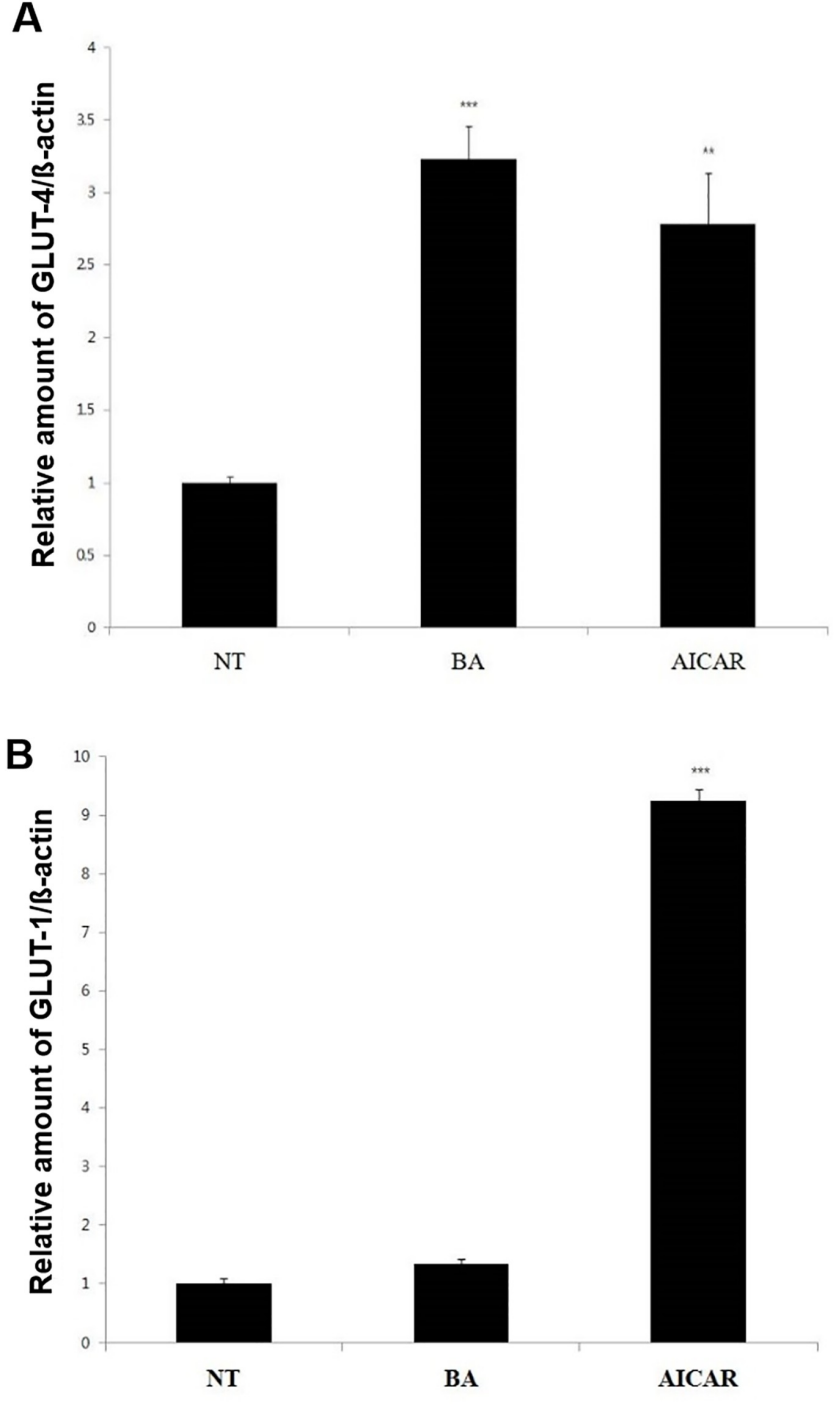
Effect of BA on mRNA expression of *Slc2a4* and *Slc2a1* genes. The mRNA expression levels of the *Slc2a4* and *Slc2a1* genes that encode glucose transporters GLUT4 (Fig 6A) and GLUT1 (Fig 6B), respectively, were measured by real-time PCR in C2C12 cells. The values represent the mean fold change ± standard error of the mean (*N* = 3). Statistical significance of differences from the control non-treated group (NT) is indicated as follows: **P < 0.01, ***P < 0.001.

To further investigate the mechanisms of increased glucose tolerance following administration of BA, we measured the mRNA expression levels of the *Slc2a4* and *Slc2a1* genes, which encode glucose transporters GLUT4 and GLUT1, respectively, in C2C12 cells. We found that BA selectively enhanced the expression of *Slc2a4* mRNA, whereas AICAR increased both *Slc2a4* and *Slc2a1* mRNA expression levels (Fig 6).

### BA effects on glucose uptake in C2C12 cells are phosphoinositide 3-kinase-independent

**Fig 7:**
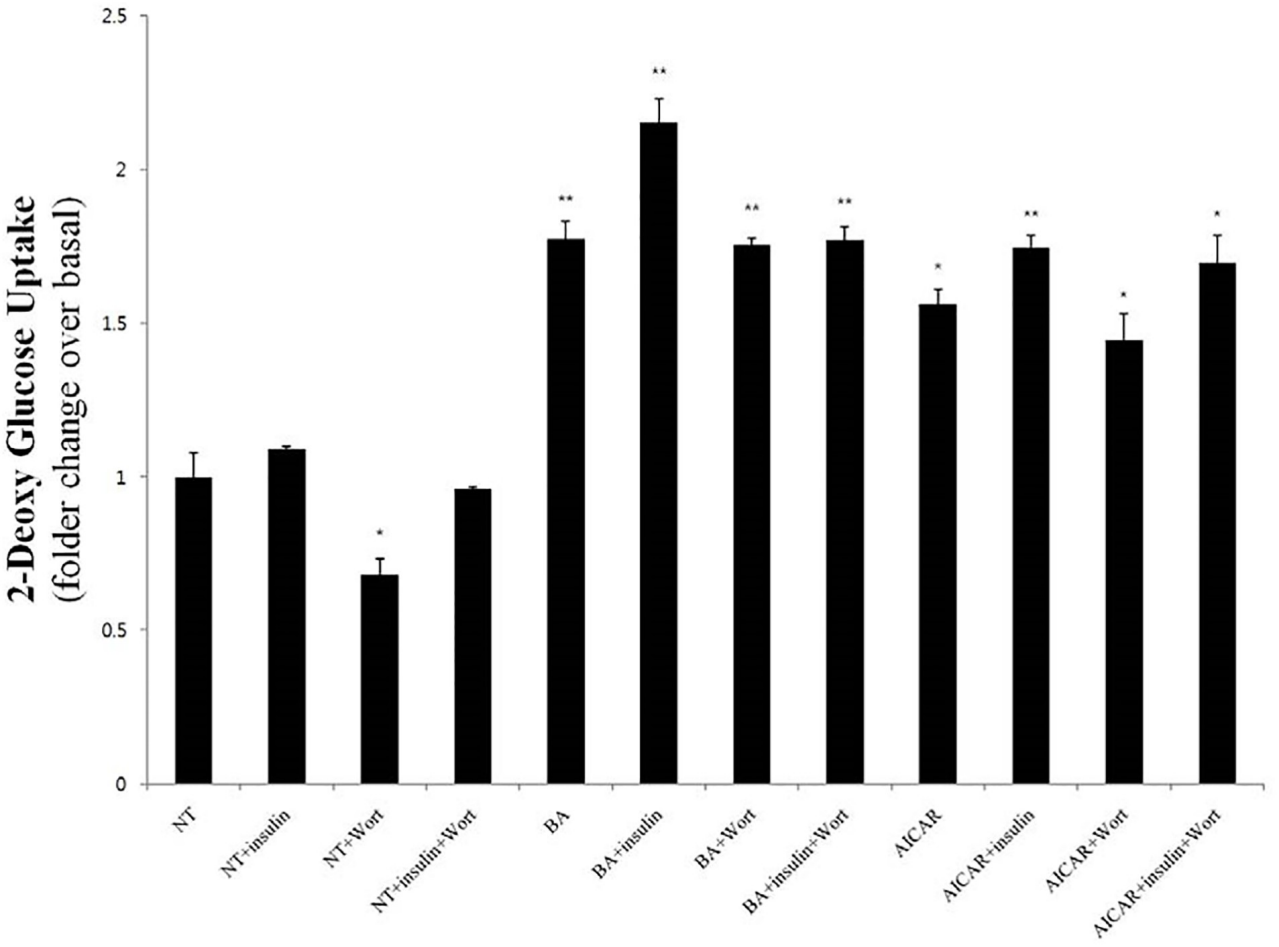
BA-induced increase of glucose uptake in C2C12 cells is independent of PI3K activity. The levels of ^3^[H]-2-deoxyglucose uptake into C2C12 cells are illustrated after their treatment with insulin, BA, or AICAR, as well as combinations thereof in the presence or absence of the PI3K inhibitor wortmannin (500 nM). The values represent the mean fold change ± standard error of the mean (*N* = 3). Statistical significance of differences from the control non-treated group (NT) is indicated as follows: *P < 0.05, **P < 0.01.

To establish whether phosphoinositide 3-kinase (PI3K) is implicated in the potentiating effect of BA on glucose uptake, we examined the latter in different experimental conditions in the presence of the PI3K inhibitor wortmannin. As shown in Fig 7, wortmannin did not significantly modify the extent of the upregulation of 2-deoxyglucose uptake compared to that afforded by BA alone. Therefore, we concluded that unlike insulin, which induces glucose uptake by the PI3K-dependent canonical pathway, BA increased glucose uptake by a different, PI3K-independent mechanism.

### BA-induced AS160 phosphorylation is PI3K/Akt-independent in skeletal muscle cells

**Fig 8:**
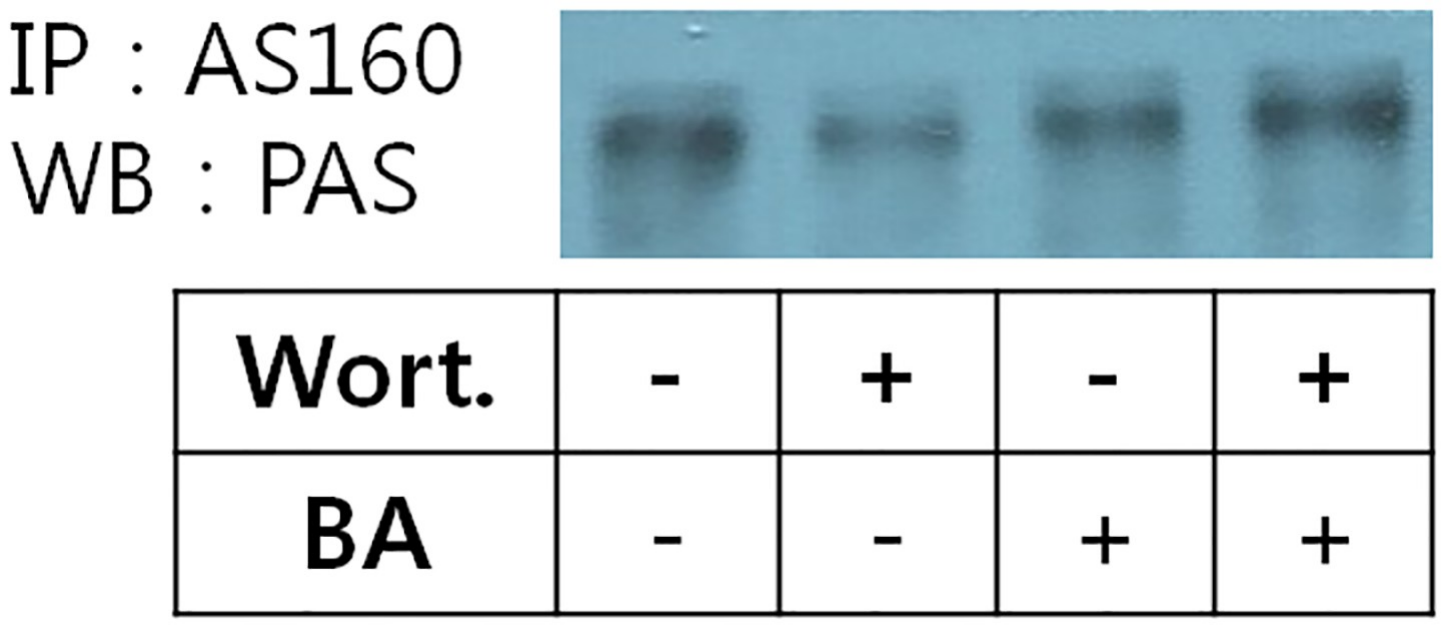
Effect of BA on AS160 phosphorylation in extensor digitorum longus muscle cells. Lysates were prepared from extensor digitorum longus muscle cells incubated for 40 min in the absence (basal) or presence of BA, wortmannin or a combination thereof. Phosphorylated AS160 was detected by western blot (WB) analysis of anti-AS160 immunoprecipitates (IP) with an anti-phosphoAS160 (PAS) antibody.

When the PI3K/Akt pathway is activated by insulin signaling, Akt phosphorylates AS160, which in turn stimulates the translocation of GLUT4 from the cytoplasmic vesicles into the cell membrane and thereby enhances the transport of glucose into the cell. To confirm that the effect of BA on glucose uptake is independent of the PI3K/Akt pathway activity, we checked the level of AS160 phosphorylation when that signaling pathway was inhibited. Previous studies have showed that isolated rat epitrochlearis muscle was exposed to AICAR, which induced increase of AS160 phosphorylation [23]. Immunoprecipitation experiments confirmed a phospho-protein of ~160 kDa was AS160 (Fig 8). We found that exposure to BA induced AS160 phosphorylation that was not sensitive to the pretreatment with wortmannin, and therefore, another pathway maybe associated with BA-induced AS 160 phosphorylation except the insulin mediated canonical signal cascade.

### BA improves forced endurance capacity

**Fig 9:**
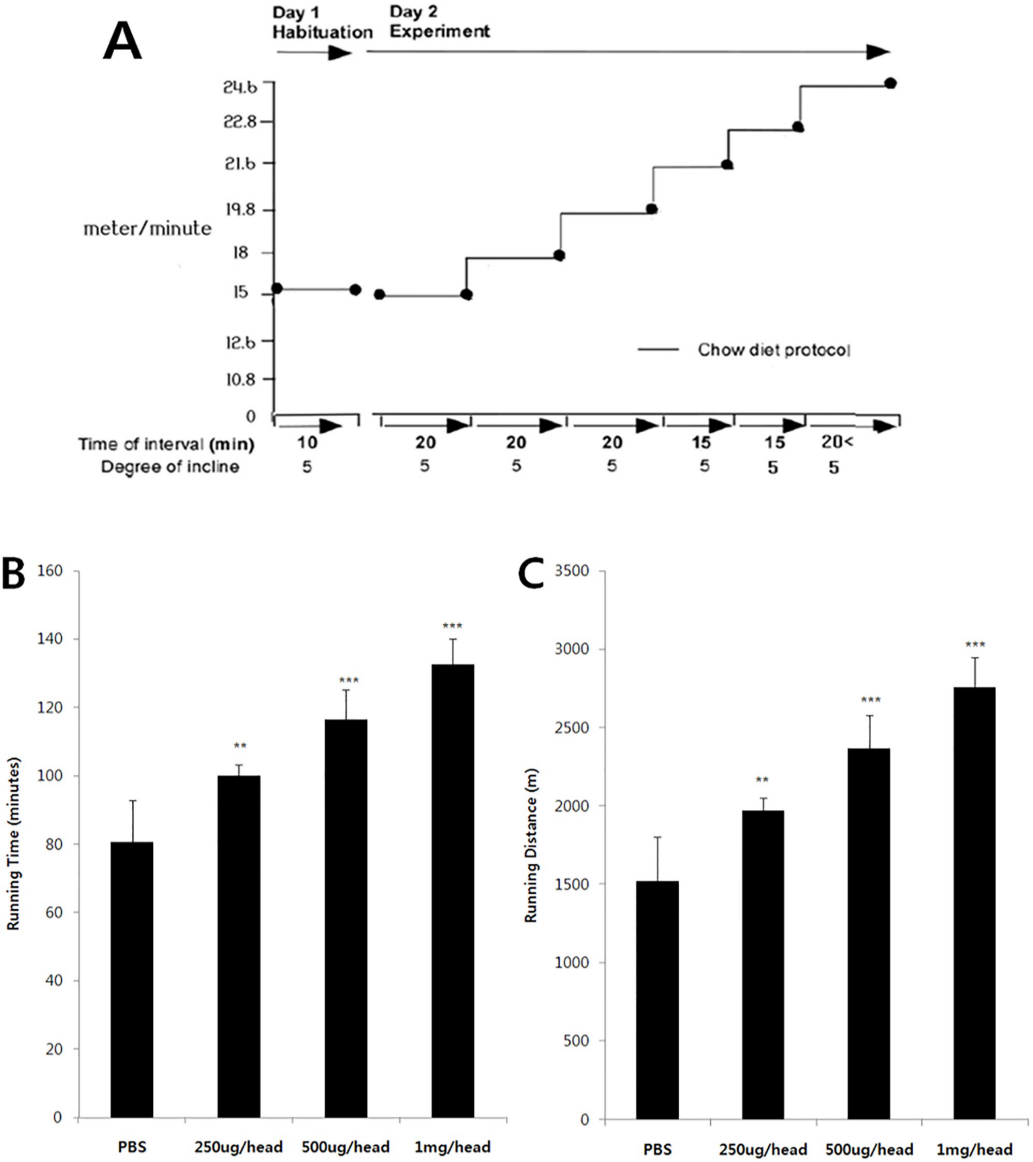
Enhanced endurance capacity in BA-treated mice during 4 weeks. (A) The protocol of the treadmill-running test used to examine the effect of BA on endurance capacity. Running time (B) and distance run (C) parameters are compared between animals treated with phosphate buffered saline (PBS) or BA at different doses. Data are presented as the mean ± standard error of the mean (*N* = 5 per group). Statistical significance of differences from the control PBS-treated group is indicated as follows: **P < 0.01, ***P < 0.001.

Increasing the activity of AMPK exerts a number of health benefits. Several studies have shown that pharmacological AMPK activation has been associated with increased exercise capacity. The effect of BA administration was further evaluated on endurance capacity of mice. Notably, in the treadmill endurance test, treatment with BA significantly prolonged running time and extended running distance compared to those in chow-fed controls (Fig 9). Thus, BA treatment significantly increased the resistance of the animals to muscle fatigue.

## Discussion

In this study, the molecular mechanism of the antidiabetic activity of BA was investigated. It was an initial hypothesis that BA possesses antidiabetic activity because it enhanced AMPK phosphorylation and, therefore, AMPK activity in C2C12 mouse muscle cells. To support this hypothesis, we demonstrated that BA decreased the level of blood glucose in alloxan-treated mice used as a diabetes model. During intraperitoneal glucose tolerance test, there was a significant increase in glucose clearance afforded by BA treatment, whereas AICAR at a dose of 200 mg/kg had only a modest effect. It also showed that these effects of BA might be at least partly mediated by the upregulation of GLUT4 expression and increased glucose uptake rate into cells, which would collectively result in the decrease of glucose level in blood. According to a recent report, AS160 is a target protein of AMPK in the antidiabetic signaling pathway. Both insulin canonical cascade and AMPK signaling could phosphorylate AS160 protein. As BA is an AMPK activator, our data on the positive effect of BA on AS160 phosphorylation were in line with the notion that AS160 is a downstream target of AMPK [24]. Whether AMPK activation by BA depended on PI3K activity was very important for understanding the molecular mechanism of AMPK activation. Adding with wortmannin, the PI3K inhibitor, did not inhibit AMPK activation induced by BA, which means that the latter proceeded in a PI3K-independent manner, as did AMPK activation by AICAR [24].

Various kinds of herbal extracts have been reported to have a preventive or a therapeutic effect on DM. During the last several years, our group has been trying to isolate principal compounds with antidiabetic activity from the Korean mistletoe extract [25]. Interestingly, BA and OA (oleanolic acid), the main ingredients of the Korean mistletoe extract, have been repeatedly shown to exhibit antitumor efficacy. It has been now well-documented that BA might be a promising anticancer drug [26]. In fact, some derivatives of BA are currently undergoing clinical trials [27,28], which means that it has great potential to be developed into an antidiabetic drug like metformin, which has both anticancer and antidiabetic properties [26]. The substances that activate AMPK usually have many diverse biological effects, including anticancer, antidiabetic, and anti-obesity effects, extension of lifespan, thermogenesis, and improved endurance capacity, like resveratrol [26,29–32]. BA may therefore be expected to have various physiological activities beyond anticancer and antidiabetic effects, thus necessitating future studies to examine these possibilities.

Herbal extracts contain many kinds of various triterpene derivatives besides BA. Several triterpenes, including BA, have been isolated from Cortex Moutan (*Paeonia suffruticosa*) and examined for the AMPK-potentiating activity, with palbinone showing stronger positive effect than BA [21]. Cucurbitine tripenoids have been recently demonstrated to be the likely major contributors to the antidiabetic effects of bitter melon [33]. Santos *et al*. [34] and Yogeeswari & Sriram [35] synthesized many derivatives of BA and examined their anticancer effects. If AMPK activation is a common feature of both anticancer and antidiabetic properties, it will be very useful to study the structure-activity relationships of triterpene derivatives in order to develop optimized leads for antidiabetic drugs.

## Supporting information

S1 Fig The informations of antibody. (TIF)

S2 Fig Gel results serves the effect of BA on AMPK phosphorylation in C2C12 cells. (TIF)
