# Supplementary figures and images for: Antidiabetic effects of betulinic acid mediated by the activation of the AMP-activated protein kinase pathway
Source: PLoS One. 2021 Apr 5;16(4):e0249109. doi: 10.1371/journal.pone.0249109 (PMC8021171; doi:10.1371/journal.pone.0249109)

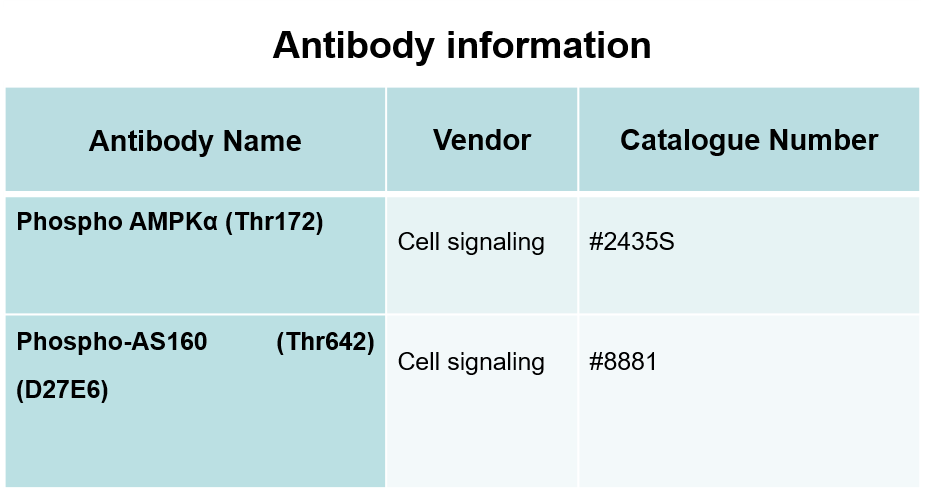

Supplement: S1 Fig — (TIF) [file pone.0249109.s001.tif]

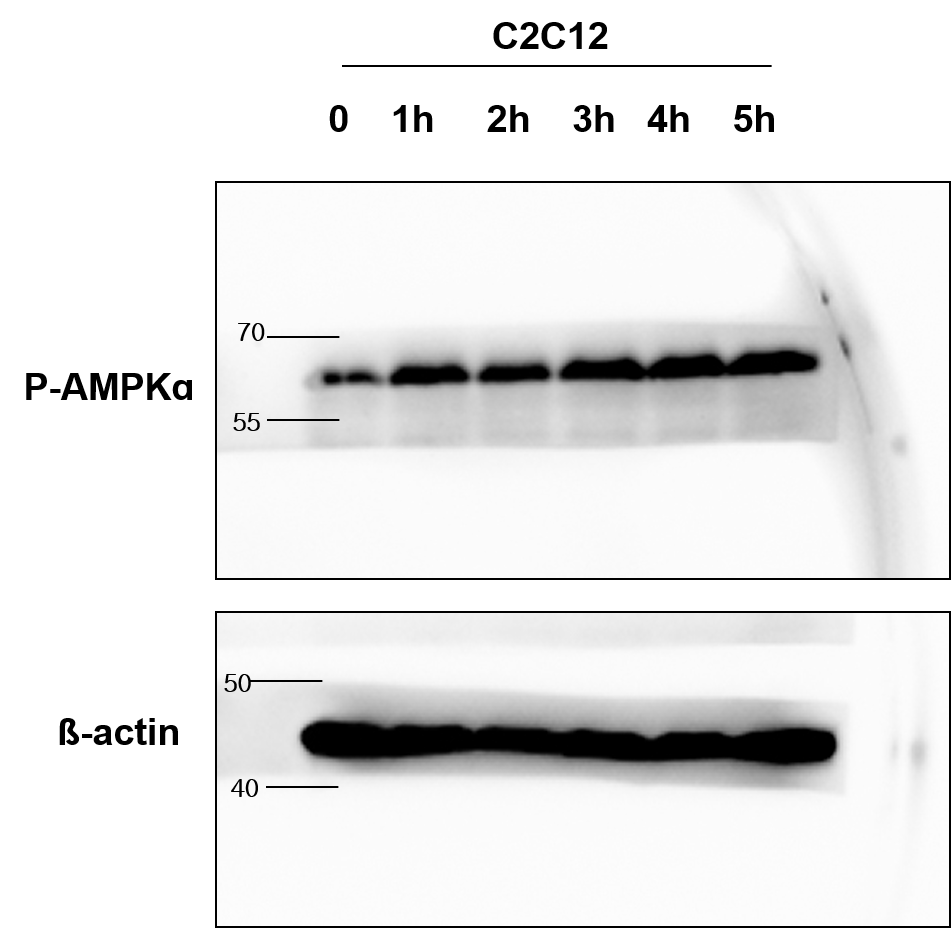

Supplement: S2 Fig — (TIF) [file pone.0249109.s002.tif]
